# Machine learning models for predicting depression in Korean young employees

**DOI:** 10.3389/fpubh.2023.1201054

**Published:** 2023-07-12

**Authors:** Suk-Sun Kim, Minji Gil, Eun Jeong Min

**Affiliations:** ^1^College of Nursing, Ewha Womans University, Seoul, Republic of Korea; ^2^Department of Medical Life Sciences, School of Medicine, The Catholic University of Korea, Seoul, South Korea

**Keywords:** machine learning, depression, employees, workplace, prediction

## Abstract

**Background:**

The incidence of depression among employees has gradually risen. Previous studies have focused on predicting the risk of depression, but most studies were conducted using basic statistical methods. This study used machine learning algorithms to build models that detect and identify the important factors associated with depression in the workplace.

**Methods:**

A total of 503 employees completed an online survey that included questionnaires on general characteristics, physical health, job-related factors, psychosocial protective, and risk factors in the workplace. The dataset contained 27 predictor variables and one dependent variable which referred to the status of employees (normal or at the risk of depression). The prediction accuracy of three machine learning models using sparse logistic regression, support vector machine, and random forest was compared with the accuracy, precision, sensitivity, specificity, and AUC. Additionally, the important factors identified *via* sparse logistic regression and random forest.

**Results:**

All machine learning models demonstrated similar results, with the lowest accuracy obtained from sparse logistic regression and support vector machine (86.8%) and the highest accuracy from random forest (88.7%). The important factors identified in this study were gender, physical health, job, psychosocial protective factors, and psychosocial risk and protective factors in the workplace.

**Discussion:**

The results of this study indicated the potential of machine learning models to accurately predict the risk of depression among employees. The identified factors that influence the risk of depression can contribute to the development of intelligent mental healthcare systems that can detect early signs of depressive symptoms in the workplace.

## Introduction

Depression in the workplace and young adults have become a growing global concern due to greater societal costs and reduced work productivity (1). In 2019, the incremental economic burden of Korean adults with depression was an estimated a total of KRW 4.83 trillion, with 14.3% in direct costs and 85.6% in indirect costs. Among indirect costs, workplace costs accounted for the highest percentage (65.6%) including absenteeism (18%) and presenteeism (42%) (2). Employees’ depression severity increased direct costs through utilization of mental health services and indirect costs attributable to overall work impairment (1, 3).

The highest prevalence of depression in young adults especially contributes to increasing an enormous economic and social burden (4). Approximately 25% of Korean younger adults aged 19–39 years experienced moderately severe depression, compared with about 18% of adults aged 40–50 years (5). However, only 7.4% of Korea employees had a diagnosis of depression by a doctor, while more than 20% had a diagnosis of depression in Canada (20.7%), United States (22.7%) and South Africa (25.6%) (6). Owing to fear of losing their job and mental health stigma, they were reluctant to disclose mental health problems and seek mental health service (6, 7).

Previous studies commonly used traditional statistical methods, such as regression analysis, to infer the relationships between depression and specific variables (8–10) which were derived from work-related theories such as the conservation of resources theory (11), self-determination theory (12), and the job demands-resources theory (13). Several studies identified factors associated with depression, including sociodemographic factors, traits, stressors, relationship stability, and cognitive processes (14, 15). In particular, work-related factors such as long working hours, workload, and burnout have been found to increase the risk of depression among employees (16, 17). However, traditional statistical methods are limited on representing real-world complexities and predicting future data due to their assumption of linearity between variables (18, 19).

Traditional statistical methods aim to test hypotheses which derive from theories, while machine learning (ML) methods focus on discovering hidden interaction in the specific data-set to make predictions (8, 9, 20). ML methods provide more accurate prediction by analyzing complex and non-linear interactions among datasets rather than separately considering the effect of one variable on an outcome of interest (21, 22). ML methods can facilitate early detection by predicting the risk of diseases (23–25). However, there were few studies on the prediction of depression using ML algorithms in young children (26), university students (27), and older adults (24).

Therefore, this study aimed to evaluate the performance the performance of different ML algorithms, such as sparse logistic regression, support vector machines (SVM), and random forest (RF), and identify the important factors influencing the risk of depression among Korean employees.

## Methods

### Data and sample

The target population were Millennial and Generation Z (MZ) employees in South Korea. This study included participants who were aged between 20 and 40; Millennials were born between 1983 to 1994, and Generation Zs were born between 1995 to 2004.

The Ewha womans university institutional review board approved this study (ewha-202206-0001-01). Potential participants were recruited from a website and social networking services during June 2022. Of the 505 employees completed the survey *via* the online survey platform, we excluded two participants (0.4%) who were over the age of 40. A total of 503 employees were used for data analysis.

### Outcome variable

The outcome variable was the depression CES-D score (28), which consists of 20 items that are rated on a 4-point Likert scale (0–3). Possible score ranges from 0 to 60, with a higher score indicating more depression-related symptoms. The Cronbach’s alpha of the CES-D was 0.85–0.90 (28) and 0.91 in the current study. Based on the CES-D cutoff score ≥ 16 (29), we divided the young employees into two groups: normal (n = 176) and at risk of depression (n = 327).

### Predictor variables

The predictor variables consisted of a set of demographics, physical health-related, job-related, and study variables that were selected based on literature reviews of the risk and protective factors for depression among employees. Study variables included personality-related variables, psychosocial protective variables, psychosocial risk variables in the workplace, and psychosocial protective variables in the workplace. Cronbach’s alpha test was used to determine inter-item reliability (Table 1). However, due to the potential for misleading results stemming from limited item variance (30, 31), Cronbach’s alpha test was not applied to measures with fewer than three items, including the 10-item Big Five Inventory (32, 33) and relationship questions (34).

**Table 1:** Predictor variables.

Factors	Variables	Indicator	Cronbach’ α
Demographic	Age		
	Gender:	Male = 0, Female = 1	
	Religion	Nonreligious = 0, Religious = 1	
	Marital status	Single = 0, Married = 1	
Physical health-related factors	Psychical activity per week		
	Amount of sleep	Less than 6 h = 0 More than 6 h = 1	
	Number of meals per day		
	Drinking	Nondrinker = 0, Drinker = 1	
	Smoking	Nonsmoker = 0, Smoker = 1	
Job-related factors	Total years of job experience		
	Employment period at their current workplace		
	Number of turnovers		
	Weekly working hours	Less than 40 h = 0 40 h = 1 From 40 to 52 h = 2 More than 52 h = 3	
	Monthly salary		
	Income satisfaction	Dissatisfied = 0 Satisfied = 1	
Psychosocial protective factors	Personality (10-item big five inventory)	Extraversion, Conscientiousness, Openness to experience, Neuroticism, Agreeableness	
	Grit (Short Grit scale)	Consistency of interest, Perseverance of effort	0.63
	Attachment (Relationship questions)	Secure, Fearful, Preoccupied, Dismissing attachment.	
	Satisfaction with life (Satisfaction with Life Scale)		0.84
	Interpersonal relationships (Relationship Change Scale)	Satisfaction, Communication, Trust, Intimacy, Sensitivity, Openness, Understanding.	0.90
Psychosocial risk factors in the workplace	Burnout (Burnout Assessment Tool)	Exhaustion, Mental distance, Emotional impairment, Cognitive impairment	0.88
	Occupational stress (Korean Occupational Stress Scale– Short Form)	Job demands, Degree of autonomy, Job instability, Organizational system, Lack of reward, Occupational climate	0.79
Psychosocial protective factors in the workplace	Occupational self-efficacy (Occupational Self-Efficacy Scale)		0.87
	Social Problem-Solving style (Social Problem-Solving Inventory-Revised-Short Form)	Positive problem orientation, Negative problem orientation, Rational problem solving, Impulsive/careless style, Avoidance style	0.76
	Meaning in work (Working and Meaning Inventory)	Positive meaning in work, Meaning-making through work, Greater good motivation	0.87
	Work-Life Balance (Work-Life Balance Scale)	Family, Leisure, Growth, Life	
	Psychological safety (Team Psychological Safety Scale)		0.54

Demographic characteristics included age, gender, religion, and marital status. Age was used as a continuous variable, while the rest of variables were used as dummy variables.

Personality-related factors included physical activity per week; the amount of sleep and number of meals per day; and drinking and smoking. The frequency of physical activity per week and the number of meals per day were used as continuous variables, while the rest of the variables were used as dummy variables.

Regarding job characteristics, we examined total years of job experience, employment period at their current workplace, number of turnovers, weekly working hours, monthly salary, and income satisfaction.

As psychosocial protective factors, we considered personality, grit, attachment, satisfaction with life, and interpersonal relationships. For personality, the 10-item Big Five Inventory (32, 33) was used. Grit was measured using the short grit scale (35), and attachment was measured by the relationship questions (34). The satisfaction with life scale (36) and relationship change scale (37) were also included.

Psychosocial risk factors in the workplace included burnout and occupational stress. Burnout was measured by the burnout assessment tool (38). Occupational stress was measured by the Korean Occupational Stress Scale-Short Form (39).

Psychosocial protective factors in the workplace included occupational self-efficacy, social problem-solving style, meaning in work, work-life balance, and psychological safety. Occupational self-efficacy was assessed using the occupational self-efficacy scale (40), social problem-solving style was evaluated using the Social Problem-Solving Inventory-Revised Short Form (41), meaning in work was measured by using the Working and Meaning Inventory (42), work-life balance was assessed using the Work-life Balance Scale (43), and psychological safety was evaluated using the Team Psychological Safety Scale (44).

With advances in data science technology, this study demonstrated the practical applicability of ML algorithms in predicting the risk of depression among MZ employees. We applied three different ML algorithms – sparse logistic regression, RF, and SVM. We found the highest accuracy of RF. Our study identified the important variables influencing the risk of depression among Korean employees such as gender, inadequate sleep, smoking habits, occupational stress, burnout, social problem-solving styles, sense of meaning at work, attachment, interpersonal relationships, and satisfaction in life. These findings contribute to the development of intelligent mental healthcare systems for the early detection of depression. Additionally, our study can help develop target interventions designed to prevent employees’ depression and provide a situation-specific theory that predicts depression among MZ employees. However, this study focuses solely on MZ employees, and thus, careful consideration is recommended before generalizing these findings to other demographic groups.

### Statistical analysis

#### Prediction models

Our goal is to predict the class of a sample given set of predictor variable values. Three different models are considered for the risk prediction: sparse logistic regression, support vector machine, and random forest. Logistic regression is one of the most widely used statistical prediction model for binary classification problem. When the response variable is binary, logistic regression predicts the probability to be classified to one of two groups given a set of covariate values. The model has the nice property that the estimated coefficients is log odds ratio. However, it is hard to interpret the results when the number of variables gets bigger. To overcome this problem, sparse logistic regression that employs least absolute shrinkage and selection operator (LASSO) in the model is considered in our problem. This model conducts feature selection and the estimation simultaneously, which enables interpretation with few selected important predictors.

Support vector machine is a famous machine learning technique for the binary classification problem. SVM seeks a decision boundary that well separate the data into two groups. It is well known that SVM performs well when data exhibit high-dimensionality while its computational cost is relatively cheap compared to other machine learning methods (45).

Random forest is the representative classification method of ensemble models, which consists of many decision trees. Ensemble method is an approach to combines prediction results from numerous algorithms to improve prediction power by avoiding overfitting. Random forest aggregates the prediction results from many decision trees to make the final decision.

To evaluate the prediction performance of above three estimated models, we used 70% of dataset for the estimation of models and last 30% of data were used for the test. To select the optimal hyperparameters in each model, 5-fold cross validation (CV) were used. For sparse logistic regression, we re-fit logistic regression using only selected features from sparse logistic regression with CV to avoid possible bias of results. Various measures including accuracy, precision, sensitivity, specificity, F1, and AUC were calculated to compare prediction performance. All statistical analyses were performed using R version 4.1.13 statistical package (R Project for Statistical Computing).

## Results

Table 2 shows the participants’ characteristics by their level of depressive symptoms. The *p*-values were computed using the t-test or chi-square test depending on the type of each variable. The results demonstrated that gender and marital status were significantly different between the normal and depression-risk groups.

**Table 2:** Socio-demographic characteristics (*N* = 503).

		Young employees’ depression		*p*-Values
		Normal range (*n* = 176, 35.0%)	Risk of depression (*n* = 327, 65.0%).	
Age	Median [1st, 3rd quartile]	32.00 [29.00, 3,500]	32.00 [30.00, 36.00]	0.204
Gender				
Male	*n* (%)	81 (46.0%)	112 (34.3%)	0.010
Female	*n* (%)	95 (54.0%)	215 (65.7%)	
Religion				
Nonreligious	*n* (%)	115 (65.3%)	220 (67.3%)	0.661
Religious	*n* (%)	61 (34.7%)	107 (32.7%)	
Marital status				
Single	*n* (%)	109 (61.9%)	245 (74.9%)	0.003
Married	*n* (%)	67 (38.1%)	82 (25.1%)	
Work related characteristics				
Total years of experience	Median [1st, 3rd quartile]	6.00 [3.00, 10.00]	5.00 [3.00, 9.00]	0.482
Employment period at their current workplace	Median [1st, 3rd quartile]	3.00 [1.00, 5.00]	4.00 [2.00, 5.00]	0.001
Number of turnovers	Median [1st, 3rd quartile]	1.00 [0.00, 2.00]	0.00 [0.00, 2.00]	0.371
Monthly salary (mln won)	Median [1st, 3rd quartile]	2.80 [2.50, 3.50]	3.00 [2.50, 3.20]	0.453

**Figure 1:**
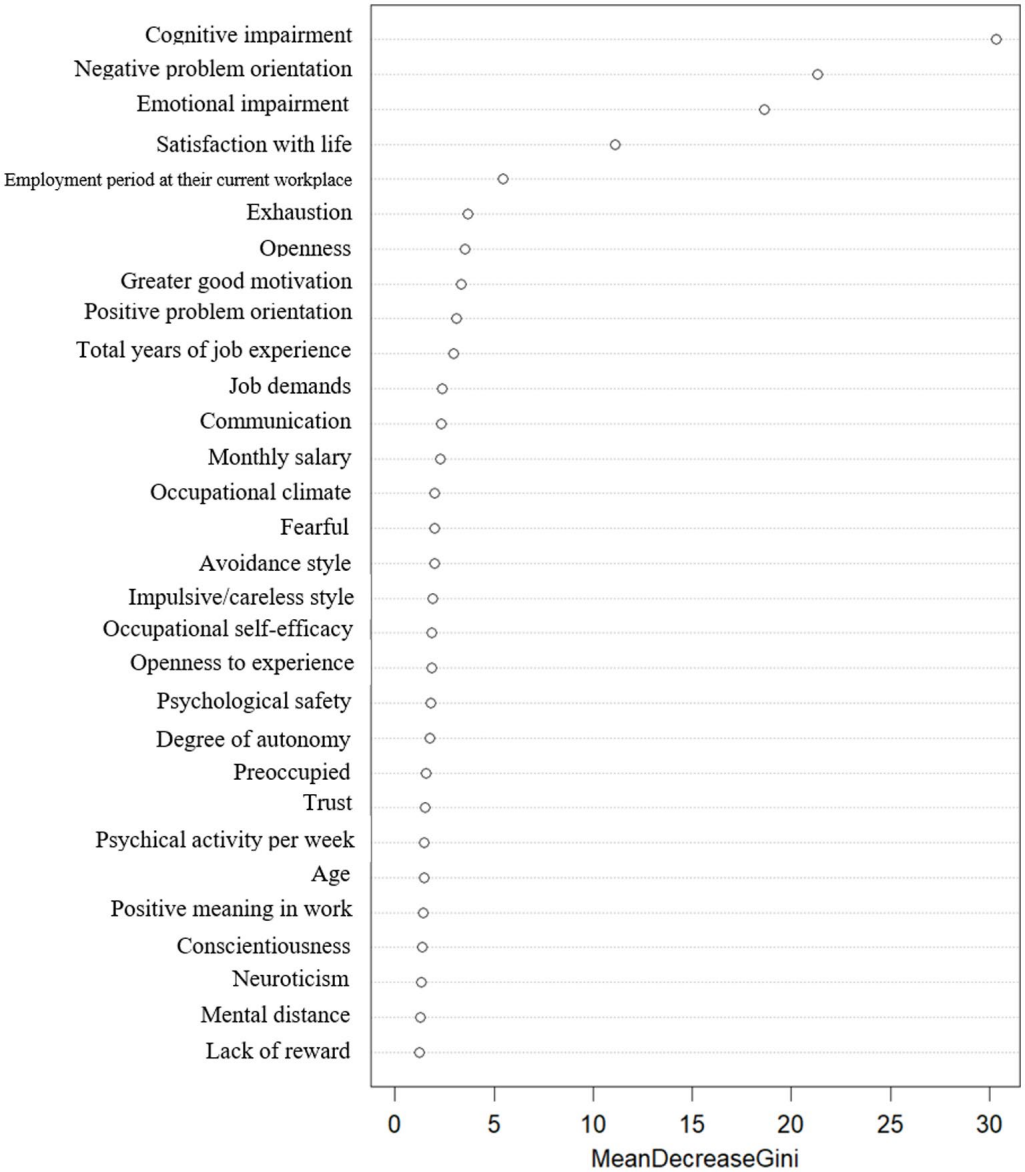
Random forest analysis results.

Performance measures calculated from analysis results of three methods are shown in Table 3. While all three models show relatively comparable performance, random forest shows little higher performance on every performance measure except AUC. This implies that random forest predicts the risk of depression more accurately. Also, random forest ranks the importance of features by comparing the reduction of average impurity on prediction for each feature. Figure 1 shows the results including first five features selected as important variables: Cognitive impairment, Negative problem orientation, emotional impairment, satisfaction with life, and employment period at their current workplace.

**Table 3:** Performance of machine learning algorithms.

	Accuracy	Precision	Sensitivity	Specificity	AUC
Sparse logistic regression	0.8675	0.8700	0.9255	0.7719	0.9171
SVM	0.8675	0.8491	0.9574	0.7192	0.8384
Random Forest	0.8874	0.8889	0.9362	0.8070	0.8716

It is worth to note that sparse logistic regression shows better performance on AUC compared to random forest. These results imply that sparse logistic regression shows better performance in terms of arrangement of samples from low depression probability to high probability. In addition to that, sparse logistic regression yields interpretable results with the estimated effect size of selected features. Table 4 includes the estimated result of logistic regression model using selected features from the sparse logistic regression. Gender, amount of sleep hours, smoking status, year of job experience, weekly working hours, interpersonal relationships, occupational stress, and social problem-solving style were shown to be important to predict the risk of depression for workers in Korea. Several features including negative problem orientation, emotional impairment is commonly shown as important features for the risk prediction in both random forest and sparse logistic regression models. Figure 1 shows significant RF variables. Burnout, social problem-solving style, and satisfaction with life were selected as important features in the RF.

**Table 4:** Logistic regression analysis results.

	Estimate	Std error	Exp coef	*p*-Value
Gender				
Male	–	–	–	–
Female	1.216	0.544	3.374	0.025
Marital status				
Single	–	–	–	–
Married	−0.836	0.579	0.434	0.149
Sleep hours				
Less than 6 h.	–	–	–	–
More than 6 h.	−1.069	0.536	0.343	0.046
Smoking status				
Non-smoker	–	–	–	–
Currently smoker	4.530	1.263	92.776	0.000
Total years of job experience	−0.227	0.081	0.797	0.005
Weekly working hours				
Less than 40 h	–	–	–	–
40 h	−1.578	0.850	0.206	0.063
From 40 to 52 h	−2.379	1.012	0.093	0.019
More than 52 h	0.690	1.701	1.994	0.685
Income satisfaction	−0.664	0.697	0.515	0.341
Personality				
Conscientiousness	−0.042	0.423	0.959	0.921
Openness to experience	0.376	0.329	1.457	0.253
Grit				
Consistency of interest	−0.624	0.439	0.536	0.155
Attachment				
Fearful	0.418	0.180	1.520	0.020
Preoccupied	0.192	0.197	1.211	0.330
Satisfaction with life	−0.115	0.062	0.891	0.065
Interpersonal relationships				
Openness	−0.211	0.098	0.809	0.031
Burnout				
Exhaustion	0.294	0.359	1.342	0.413
Emotional impairment	1.493	0.486	4.451	0.002
Cognitive impairment	0.368	0.418	1.446	0.378
Work and meaning				
Greater good motivation	−0.256	0.141	0.774	0.071
Occupational stress				
Job demands	0.034	0.017	1.035	0.042
Social Problem-Solving style				
Negative problem orientation	0.240	0.087	1.271	0.006
Work-Life Balance				
Work-Family Balance	−0.026	0.227	0.975	0.909
Work-Growth Balance	−0.296	0.222	0.744	0.182

## Discussion

This study provides evidence that the ML algorithms can help reduce bias and accurately predict the likelihood of depression among MZ employees. The main strength of this study was the use of the MZ employees’ dataset to predict and identify personal and work-related factors of depression using ML techniques. MZ employees exhibited unique depression symptoms, such as narcissistic tendencies, a feeling of victimization from supervisors, difficulty accepting criticism, and an inferiority complex (46). Our study applied to advanced ML techniques to improve intelligent mental healthcare systems which will be used to detect early depressive symptoms and increase access to mental health services for MZ employees in Korea.

The primary objective of this study is to develop ML algorithms to predict the risk of depression among MZ employees. This objective differs from hypothesis testing, which seeks statistically effective variables in relationship with response variables. In this case, too small sample size leads to low power of the test, which means inefficient use of resources including data and time. To overcome the problem, often optimal sample size calculation for getting enough power based on the expected type I and II error of hypothesis test can be conducted (47). This approach can be wildly found in randomized control trials (RCT), especially clinical trials.

On the other hands, our study is focused on developing the predictive model, and we assess their performance using several prediction performance measures such as sensitivity and specificity. To assess the generalizability of their prediction power, ML typically uses partial portion of the data called the training dataset, and test their prediction performance using the data unused for the model construction, called the test dataset. The test set is used to get the accuracy of the ML algorithms and assess model performance regardless of sample size (10, 48). This approach enables us to estimate a prediction error (generalizability) on new data. In our study, the dataset was divided into a training set (70%) and a testing set (30%). Also, cross-validation method the we employed for ML model training is one of the representative devised method to overcome the situation with not enough sample size situation.

Machine learning techniques, sparse logistic regression, SVM, and RF, were applied to develop models for predicting the risk of depression among MZ employees. In this study, sparse logistic regression, SVM, and RF techniques yielded very close accuracies, with RF being slightly higher. Feature selection performed using sparse logistic regression and RF showed similar variables as the important factors of the risk of depression. Sparse logistic Regression provided interpretable results *via* feature selection procedure with the estimated effect size and *p*-values for testing its significance, while RF gives a comprehensive view of variable importance through impurity reduction (49, 50). SVM also showed comparable prediction performance, but also limitation of the method is clear that it does not yields any results regarding importance of each feature for prediction. The important variables identified by Sparse logistic Regression and RF could be useful as a selection tool for mental health professionals to identify employees at risk of depression.

We found that female employees were more likely to suffer from depression than male employees. This result in consistent with previous studies which reported higher levels of depression among female employees (51–53). Although the mechanisms that underlie this gender difference remain unclear, one possible explanation is related to sex-specific factors. A reduction in estrogen levels may contribute to an increased risk of depression among women (54). Moreover, female employees in East Asia face heavier domestic workloads, including housework and childcare than males, which may contribute to their depression (2, 55). These results highlight the need to identify the mechanisms underlying depression among female employees and develop tailored interventions to address their needs.

Our finding indicates that employees who sleep less than six hours per night were at a higher risk of depression than those who sleep for more than six hours. This is consistent with previous studies which reported that short sleep was associated with a performance of works and depression (56, 57). Our result suggests that adequate sleep is crucial for preventing depression among employees. Additionally, smokers were more likely to suffer from depression than non-smokers in this study. This result was consistent with previous studies that depression was associated with current smoking (58, 59). Chronic nicotine exposure can affect neurotransmitters such as dopamine and 5-HT, leading to depression (60, 61). To successfully prevent the risk of young employees’ depression, smoking cessation and preventive interventions must be developed.

In our study, occupational stress and burnout were identified as psychosocial risk factors in the workplace pivotal in predicting depression risk in young generation employees. These findings were in similar with the results from prior studies, which reported that work-related stress was associated with higher depression in employees (4, 62). A logistic regression analysis also showed that higher occupational stress from highly demanding jobs were more likely to lead to depression. However, employees whose years of job experience were longer and weekly work hours were between 40 to 52 h showed a lower risk of depression. A potential explanation is that employees who face high job demands such as high workloads, time pressure, and long working hours may tend to develop the risk of depression by feeling hopelessness and powerlessness at work when they have little or no control over their work (63–66).

Moreover, higher burnout from emotional impairment was a predictor in both Logistic Regression and RF approach. This supports the results of previous studies showing that individuals with major depressive disorder struggle with regulating emotions due to a dysfunction of their emotional brain systems (67, 68). This result can be explained that employees who experience difficulty in understanding, recognizing, and controlling emotions may undergo decreased contextual information and memory processing that subsequently impairs the autonomic nervous system and brain structure, which results in depression (67, 69, 70). Consistent with this notion, an emotional regulation program such as mindfulness and Emotional Focused Therapy (EFT) is needed to prevent young employees’ depression.

We discovered a social problem-solving style and meaning in work as significant psychosocial protective factors in the workplace for predicting employees’ depression. According to earlier studies, the deterioration of social problem-solving was associated with depression (71). Negative interpretations of the work environment and events tend to be likely to increase depressive rumination as individuals recall more negative past experiences. In contrast, a greater sense of meaning in work predicted lower depression and higher psychological well-being. Employees who have a desire to help others and contribute to society experienced fewer symptoms of depression (72, 73). To reduce depression, intervention can help MZ employees view their work as meaningful, understand challenges, and develop abilities to deal with stress and difficulties at work.

Three psychosocial protective factors, including attachment, interpersonal relationships, and satisfaction in life, were identified in predicting depression in young employees. Young employees with fearful attachments were more likely to become depressed, while young employees with interpersonal openness were less likely to become depressed. Employees with fearful attachment likely have a negative view of themselves and others causing social isolation and loneliness, but those who are confident in self-expression and have higher self-esteem feel socially connected to others which results in preventing depression (74, 75).

The current study has some limitations. First, cross-sectional data restricts the interpretation of causal relationships. Secondly, the sample was limited to young Korean young employees, making it difficult to generalize to older employees, those at different career stages, and individuals from other cultural backgrounds. Finally, the RF approach identified several important predictive factors, but the direction of effect is unclear.

## Conclusion

With advances in data science technology, this study demonstrated the practical applicability of ML algorithms in predicting the risk of depression among MZ employees. We applied three different ML algorithms – sparse logistic regression, RF, and SVM. We found the highest accuracy of RF. Our study identified the important variables influencing the risk of depression among Korean employees such as gender, inadequate sleep, smoking habits, occupational stress, burnout, social problem-solving styles, sense of meaning at work, attachment, interpersonal relationships, and satisfaction in life. These findings contribute to the development of intelligent mental healthcare systems for the early detection of depression. Additionally, our study can help develop target interventions designed to prevent employees’ depression and provide a situation-specific theory that predict depression among MZ employees. However, this study focuses solely on MZ employees, and thus, careful consideration is recommended before generalizing these findings to other demographic groups.

## Data availability statement

The original contributions presented in this study are included in the article/Supplementary material, further inquiries can be directed to the corresponding author.

## Ethics statement

The studies involving human participants were reviewed and approved by Ewha Womans University Institutional Review Board. Written informed consent for participation was not required for this study in accordance with the national legislation and the institutional requirements.

## Author contributions

S-SK, MG, and EM performed the study and manuscript conceptualization and contributed to methods, results, and discussion. S-SK and MG contributed to the background. All authors contributed to the article and approved the submitted version.

## Funding

This study was supported by Basic Science Research Program through the National Research Foundation of Korea (NRF) funded by the Ministry of Science, ICT and Future Planning (No. NRF 2022R1A2C2004867, 2022R1A6A3A01086554, and 2021R1F1A1058613).

## Conflict of interest

The authors declare that the research was conducted in the absence of any commercial or financial relationships that could be construed as a potential conflict of interest.

## Publisher’s note

All claims expressed in this article are solely those of the authors and do not necessarily represent those of their affiliated organizations, or those of the publisher, the editors and the reviewers. Any product that may be evaluated in this article, or claim that may be made by its manufacturer, is not guaranteed or endorsed by the publisher.
